# A transcriptomic view of the ability of nascent hexaploid wheat to tolerate aneuploidy

**DOI:** 10.1186/s12870-020-2309-6

**Published:** 2020-03-04

**Authors:** Deying Zeng, Jiantao Guan, Jiangtao Luo, Laibin Zhao, Yazhou Li, Wenshuai Chen, Lianquan Zhang, Shunzong Ning, Zhongwei Yuan, Aili Li, Youliang Zheng, Long Mao, Dengcai Liu, Ming Hao

**Affiliations:** 1 State Key Laboratory of Crop Gene Exploration and Utilization in Southwest China, Sichuan Agricultural University at Chengdu, Wenjiang, 611130 Sichuan China; 2 Triticeae Research Institute, Sichuan Agricultural University at Chengdu, Wenjiang, 611130 Sichuan China; 3 Institute of Crop Science, Chinese Academy of Agricultural Sciences, Beijing, 100081 China; 4 Crop Research Institute, Sichuan Academy of Agricultural Science, Chengdu, 610066 Sichuan China

**Keywords:** Homoeolog buffering, Aneuploidy, Polyploid, Synthetic wheat

## Abstract

**Background:**

In contrast to most animal species, polyploid plant species are quite tolerant of aneuploidy. Here, the global transcriptome of four aneuploid derivatives of a synthetic hexaploid wheat line was acquired, with the goal of characterizing the relationship between gene copy number and transcript abundance.

**Results:**

For most of the genes mapped to the chromosome involved in aneuploidy, the abundance of transcripts reflected the gene copy number. Aneuploidy had a greater effect on the strength of transcription of genes mapped to the chromosome present in a noneuploid dose than on that of genes mapped elsewhere in the genome. Overall, changing the copy number of one member of a homeologous set had little effect on the abundance of transcripts generated from the set of homeologs as a whole, consistent with the tolerance of aneuploidy exhibited by allopolyploids, whether in the form of a chromosomal deficit (monosomy) or chromosomal excess (trisomy).

**Conclusions:**

Our findings shed new light on the genetic regulation of homeoallele transcription and contribute to a deeper understanding of allopolyploid genome evolution, with implications for the breeding of polyploid crops.

## Background

The result of aneuploidy, whether it is due to the gain or loss of a whole chromosome or chromosome segment, is a nonstandard dosage of all or some of the genes mapped to the chromosome involved, respectively. In most diploid species, particularly in the animal kingdom, the condition typically has drastic consequences for the organism’s fitness and/or reproductive ability [1, 2]. In humans, for example, of the 22 potential autosomal trisomies, the only one that is nonfatal is trisomy 21 (Down syndrome); autosomal monosomy in mammals typically results in fetal abortion [3]. In contrast, aneuploidy is tolerated by many plant species; thus, it has been almost a century since a full set of *Datura stramonium* trisomies was isolated [4]. Polyploid species, which are very common in the plant kingdom but rare in the animal kingdom, are particularly tolerant of aneuploidy. Perhaps the best studied example of this phenomenon is presented by the hexaploid species *Triticum aestivum* (bread wheat), for which Sears [5] was able to derive an almost complete set of nullisomic, monosomic, trisomic, tetrasomic and ditelosomic variants based on the discovery of monosomic and trisomic plants among the offspring of two haploid progeny of a Chinese Spring (CS) wheat × cereal rye cross [6].

Polyploids can have a superior tolerance of aneuploidy, given their increase upon polyploidization. Studies on divergent plant taxa have demonstrated that newly created polyploid plants appear to experience a high degree of cytogenetic instability, accompanied by a high frequency of aneuploidy [7–14]. For instance, aneuploidy was found to vary from 20 to 100% among 16 new synthetic hexaploid wheat (SHW) lines [15]. SHW is created by the whole-genome doubling of hybrids between tetraploid wheat (*Triticum turgidum,* AABB genome) and diploid *Aegilops tauschii* (DD genome), somewhat duplicating the origin of bread wheat [16, 17]. SHW lines have been successfully used to enhance wheat yields across a diverse range of environments [18–21].

Studies on nascent polyploid plants can provide critical information that has implications for the genetic control of gene expression in the early stages of ancestral polyploidization events. Such data are also important for utilizing nascent polyploids in breeding and may even provide clues for cancer research, as many cancer cells are found to be aneuploids [22, 23]. The transcriptomic impact of aneuploidy in nascent polyploid plants has been scarcely documented to date, although the frequent aneuploidy in nascent polyploids allows the acquisition of true sibling plants with varied copies of chromosomes. In the experiment described here, which was undertaken to address the transcriptomic effect of aneuploidy, both the tendency of the early generations bred from a newly synthesized wheat allopolyploid to produce aneuploids and the power of the RNA-Seq platform to rapidly acquire a genome-wide transcriptome were leveraged. The outcome of the resulting transcriptomic analysis sheds new light on the regulation of homeolog transcription and deepens our understanding of allopolyploid genome evolution, with implications for the breeding of polyploid crop species.

## Results

### Karyotypic verification of aneuploidy and the effect of aneuploidy on phenotypes

**Fig. 1:**
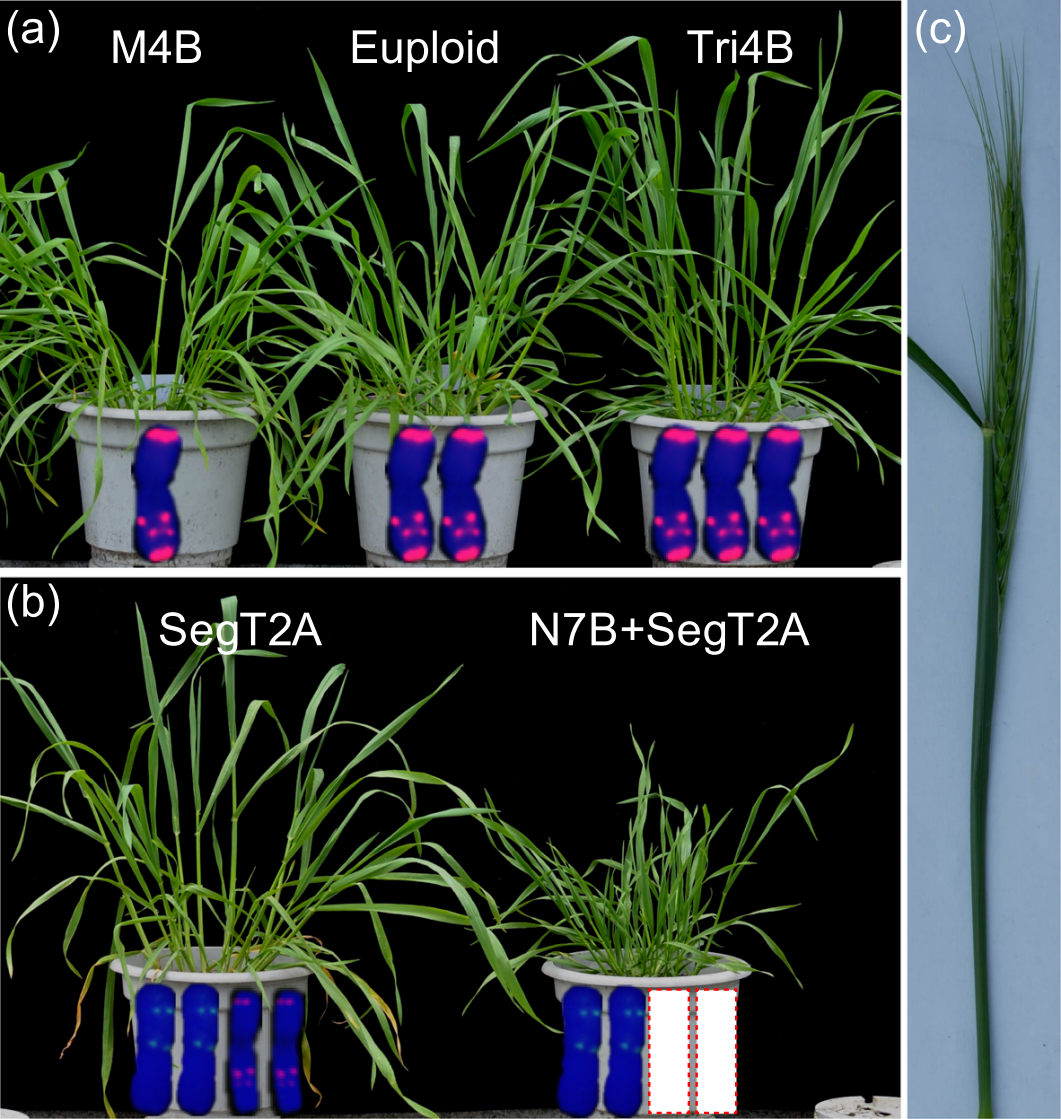
Newly synthesized hexaploid wheat. (**a**) M4B: plant lacking one copy of chromosome 4B, Tri4B: plant carrying an extra copy of chromosome 4B. (**b**) SegT2A: plant harbouring a duplicated segment of the short arm of chromosome 2A. N7B + SegT2A, plant lacking both copies of chromosome 7B (rectangle with red dotted border) and harbouring a duplicated segment of the short arm of chromosome 2A. (**c**) A spike imaged at the heading stage, illustrating the nature of the samples taken for RNA extraction

The aneuploids isolated among the progeny of the hybrid AS313 x AS60 allohexaploid comprised the following (Fig. 1): a 2n = 41 plant lacking one copy of chromosome 4B (M4B), a 2n = 43 plant trisomic for chromosome 4B (Tri4B), a 2n = 42 plant harbouring four copies of a segment of chromosome arm 2AS (SegT2A) and a 2n = 40 plant lacking both copies of chromosome 7B, along with the same altered version of chromosome 2A carried by SegT2A (N7B + SegT2A). Euploid (2n = 42) sib plants were retained as a control. The fluorescent in situ hybridization (FISH) karyotypes of these plants are shown in Figs. S1 and S2.

The loss of a copy of chromosome 4B (M4B) resulted in the formation of a narrowed third seedling leaf and lowered fertility, while in the presence of an extra copy of this chromosome (Tri4B), the spike was shortened and bore fewer spikelets than the euploid spike. SegT2A plants produced spikes that were longer than the euploid ones. The phenotype of the N7B + SegT2A plant was very different from that of the euploid, being substantially shorter, less vigorous and less fertile (Tables 1 and S1).

**Table 1:** Phenotypic performance of the aneuploid plants

Genotype	No. of plants	Chromosome constitution	Plant height (cm)	Spike length (cm)	No. of spikelets	Seed set (%)
M4B	10	20″ + 4B’	88.05 ± 2.89	17.49 ± 0.56	23.90 ± 0.48	54.49 ± 2.71*
Euploid	28	21″	86.07 ± 2.97	17.77 ± 0.39	24.23 ± 0.31	63.83 ± 2.85
Tri4B	5	20″ + 4B”’	79.76 ± 1.97	15.12 ± 0.89**	22.40 ± 0.51*	61.14 ± 2.45
SegT2A	5	20″ + Seg2A”	92.42 ± 3.38	21.14 ± 0.96*	25.40 ± 0.40	71.17 ± 3.61
N7B + SegT2A	11	19″ + Seg2A”	72.91 ± 3.06**	17.14 ± 0.34**	21.27 ± 0.36**	28.63 ± 3.06**

*, **: means differ from one another at *P* ≤ 0.05 and ≤ 0.01, respectively (statistical significance was determined using Student’s *t* test). Comparisons were made between euploid plants and the aneuploids M4B, Tri4B and SegT2A and between N7B + SegT2A and SegT2A

### RNA-Seq profiling

The RNA used for the transcriptomic analysis was extracted from spikes sampled at the heading stage (Fig. 1 b). Three or four individual plants were used as biological replicates. The RNA-Seq reads acquired covered ~ 87 × 10^9^ nt (Table S2). When mapped with IWGSC RefSeq v1.0, the average rate of unique alignment was 81.9% (range: 78.8–83.4%). The fragments per kilobase of gene per million mapped reads (FPKM) values were strongly correlated between the biological replicates (mean R^2^ = 0.94, see Table S2).

### The correlation between gene dosage and transcript abundance

**Fig. 2:**
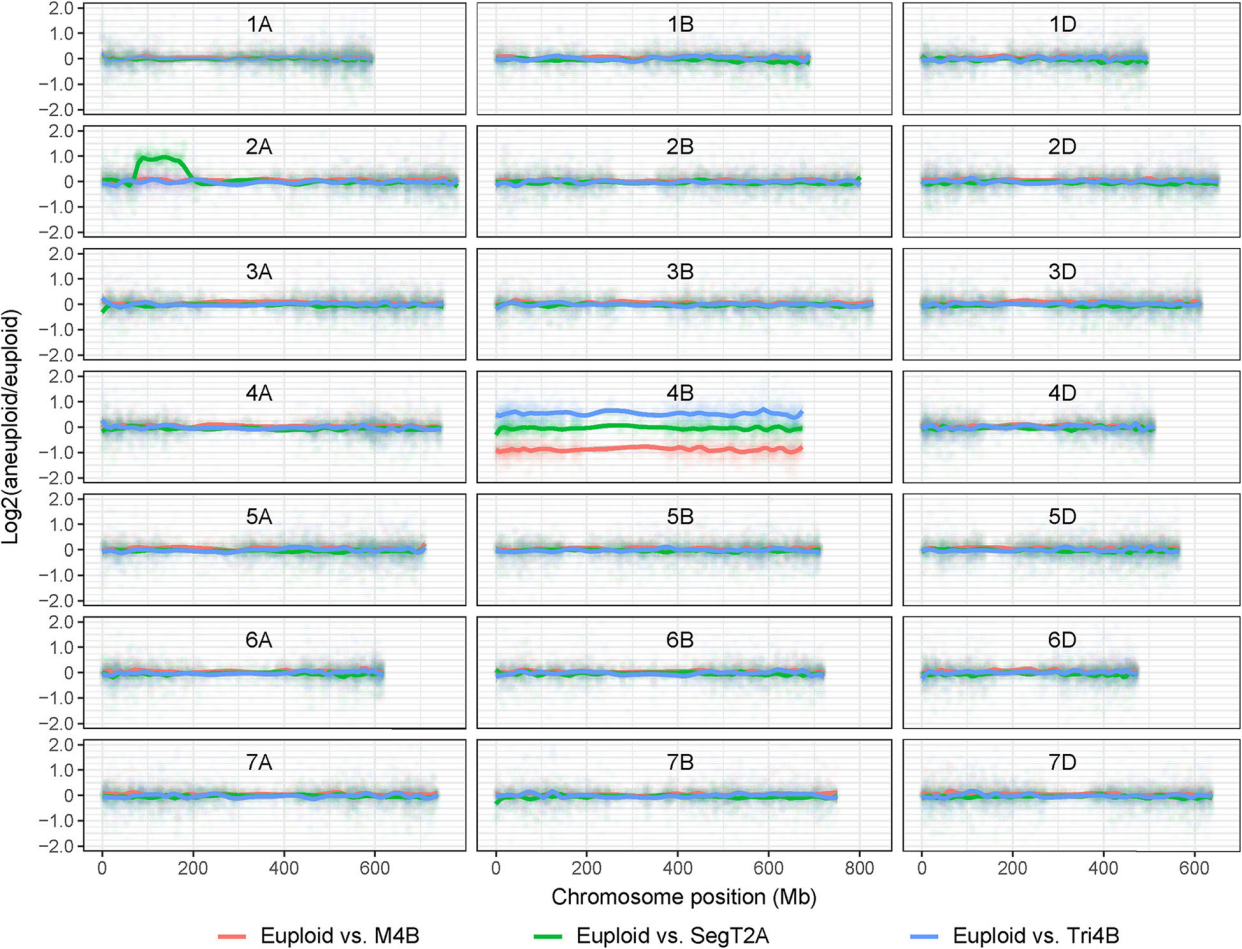
Distributions of transcription level ratios between aneuploid and euploid plants. The log_2_(aneuploid/euploid) ratios of each of 50,996 genes with an FPKM values above unity in euploids and aneuploids were plotted as a ratio distribution along each chromosome. The *x* axes correspond to the physical position of the genes. Each point represents a gene: red indicates the contrast of euploid vs M4B, blue indicates euploid vs Tri4B and green indicates euploid vs SegT2A. The correspondingly coloured lines track the smoothed distribution of the log_2_(aneuploid/euploid) ratios. A relative change in transcript abundance of zero is designated by the ratio 0 (log_2_1); a 0.5-fold increase, by 0.6 (log_2_1.5); a 0.5-fold decrease, by the ratio − 1 (log_2_0.5); and a 1.0-fold increase, by the ratio 1 (log_2_2)

The effect of aneuploidy on the global transcriptome was initially quantified by generating fold change [log_2_(aneuploid/euploid)] distributions for each of 50,996 genes with an FPKM value above unity. A clear conclusion was that genes mapped to the chromosome present in a noneuploid dose were, for the most part, transcribed at a level proportional to their copy number (Fig. 2). Thus, compared with the euploid level of transcription of genes mapped to chromosome 4B, the level in the Tri4B plant was ~ 0.5-fold higher, and that in the M4B plant was ~ 0.5-fold lower. Similarly, the 806 genes mapped within the segment of chromosome 2A involved in the SegT2A duplication were transcribed approximately twice as abundantly in SegT2A plants than in euploid plants; additionally, for the chromosome 2A genes lying outside of the duplicated segment, there was little evidence for any effect of the aneuploid condition on their transcription intensity (Fig. 2).

A Pearson correlation test conducted to estimate the proportion of genes mapped within the aneuploid chromosome/chromosome segment revealed a dosage-related alteration of their transcription level. Of the 2171 genes identified on chromosome 4B, the transcript abundance of 1862 (85.8%) was positively correlated with gene dosage. A similar calculation with respect to the 465 genes mapped within the duplicated chromosome 2A segment showed that 360 (77.4%) were transcribed at a level correlated with the segment’s dosage.

### Aneuploidy had a greater effect on the transcription of genes on the affected chromosome than on those mapped elsewhere

The set of differentially transcribed genes (DTGs) identified between the aneuploid and euploid plants was used to compare the effect of aneuploidy on the transcription of genes mapped within the affected chromosome/chromosome segment to that of genes mapped elsewhere. The transcriptome of N7B + SegT2A was not included in this analysis because of its nullisomy for chromosome 7B. The comparisons based on the transcriptomes of M4B, Tri4B and the euploid indicated that the monosomic condition induced a greater perturbation in the global transcriptome than did the trisomic condition: in M4B, 1104 genes were classified as DTGs, whereas the number in Tri4B was only 236 (Table 2), indicating an obvious effect of relative gene dosage (2 for euploid/M4B; 1.5 for Tri4B/euploid). Of the DTGs identified in the M4B vs euploid contrast, 1029 (93.2%) mapped to chromosome 4B, as did 225 (95.3%) DTGs identified in the Tri4B vs euploid contrast. Of these DTGs, 186 were shared by the two contrasts, downregulated in M4B and upregulated in Tri4B. For the chromosome 2A genes analysed using the SegT2A vs euploid contrast, 265 (80.1%) of the 331 DTGs mapped to chromosome 2A.

**Table 2:** Chromosomal distribution of DTGs

Comparison	Expressed genes	Regulation	Number of differentially expressed genes																						
			1A	1B	1D	2A	2B	2D	3A	3B	3D	4A	4B	4D	5A	5B	5D	6A	6B	6D	7A	7B	7D	Un^*^	Total
Euploid vs M4B	50,960	Down	4		2	3	3	3	1	1	1	2	1029	5		5	2	3	1	0	1	2	2	12	1082
		Up	1		2	1	2	1	1	1	0	2	–	0	2	2	1	4	0	0	1	1	0	0	22
Euploid vs Tri4B	51,304	Down	1	0	0	0	2	1	0	0	0	0	–	0	0	1	0	0	0	0	0	0	2	0	7
		Up	0	0	0	0	0	0	0	0	0	1	225	0	0	0	2	0	0	0	0	0	0	1	229
Euploid vs SegT2A	50,796	Down	0	1	0	2	4	3	0	0	3	2	4	1	2	2	1	6	4	3	2	8	2	0	50
		Up	1	0	0	263	1	1	1	0	2	1	1	2	0	1	0	0	0	2	1	2	2	0	281
SegT2A vs N7B + SegT2A	51,061	Down	39	30	39	60	51	59	47	42	54	45	37	48	54	52	49	44	34	41	56	–	60	23	964
		Up	23	31	17	34	54	42	39	48	43	31	33	28	23	42	27	27	42	24	43	–	41	11	703

*, unassigned scaffolds

Among the DTGs not mapped to the chromosome/chromosome segment involved in aneuploidy, most were downregulated as a result of the noneuploid condition (Table 2). For example, the contrast between the transcriptomes of N7B + SegT2A and SegT2A identified 1667 DTGs mapped to chromosomes other than 7B (3.3% of the transcribed set of 51,061 genes); of these, 904 were transcribed at a lower level in N7B + SegT2A, while only 763 were transcribed at a higher level. Similarly, a larger number of non-chromosome 2A DTGs were downregulated rather than upregulated in SegT2A (48/66), as was the case for the non-chromosome 4B DTGs in M4B (53/75). When a Gene Ontology (GO) analysis was conducted on these three sets of DTGs, it appeared that those with specific products involved in oxidation-reduction were well represented (Table S3). In the set derived from the N7B + SegT2A vs SegT2A contrast, genes that were downregulated were overrepresented in the response to photosynthesis, whereas those that were upregulated were overrepresented in the response to stress. There was no evidence that these DTGs were more likely than not to be homeoalleles of those affected by the aneuploid condition (Table 2).

### The buffering effect of homeoalleles

**Fig. 3:**
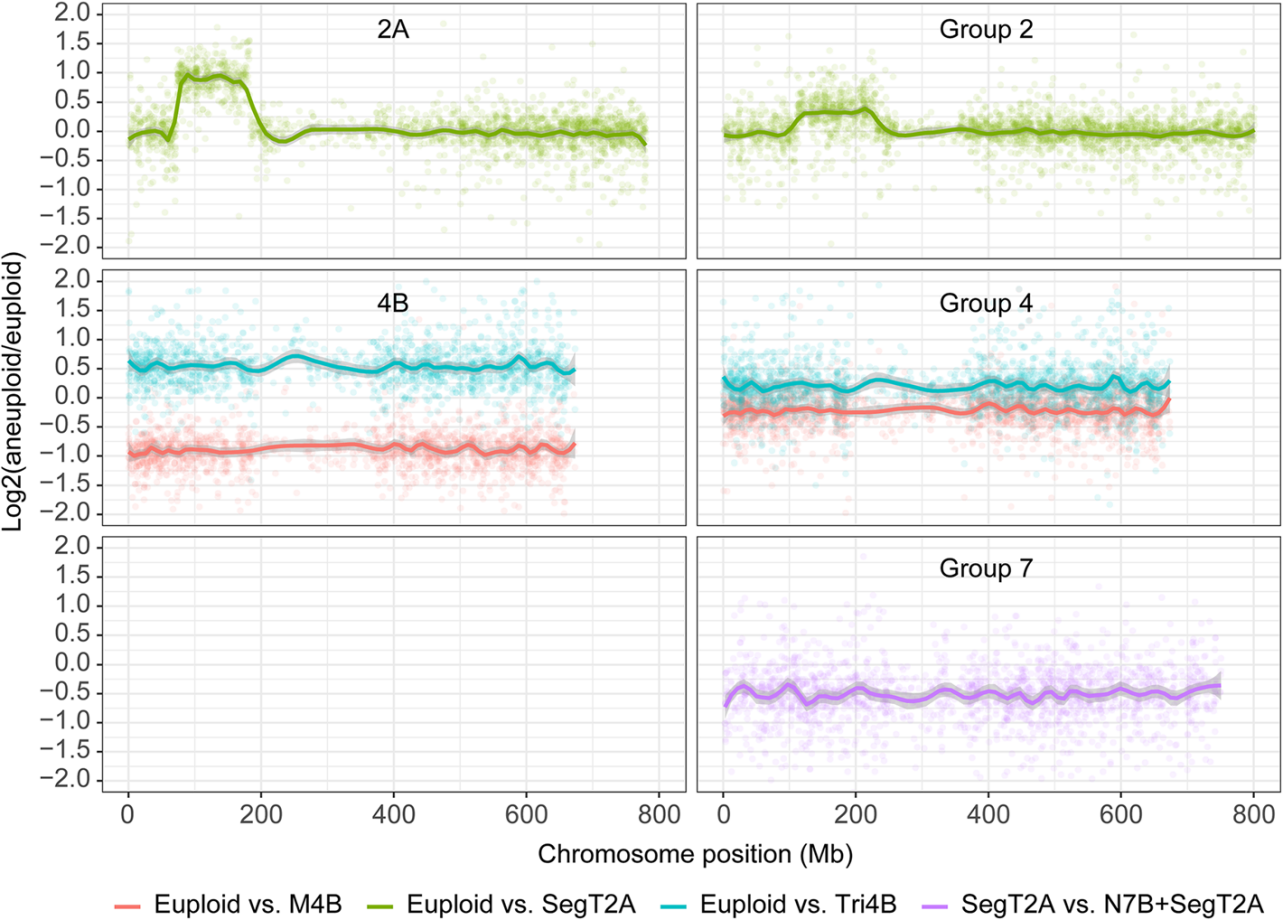
Distributions of transcription level ratios for triad genes between aneuploid and euploid plants. Left-hand panels: log_2_(aneuploid/euploid) distributions for chromosomes 4B and 2A. Right-hand panels: log_2_(aneuploid/euploid) distributions for triads of homeologous groups 2, 4 and 7. The *x* axes in the right-hand panel correspond to the physical position of genes mapped to the B subgenome

Single-copy genes are generally represented in hexaploid wheat by a triad of homeoalleles. Analysis of the global transcriptome showed that 1748 such triads were generated from the chromosomes of homeologous group 4; 2102 from the chromosomes of homeologous group 7; and 455 from the duplicated segment of chromosome 2A (Table 3). Consistent with the analysis based on the full transcriptome (Fig. 2), the level of transcription of homeoalleles of triad genes mapped to the chromosome/chromosome segment involved in aneuploidy was generally proportional to their copy number (1:2:3 for the M4B vs euploid vs Tri4B contrast and 1:2 for the euploid vs SegT2A contrast) (Fig. 3). However, the total expression level of the homeoalleles of triads in aneuploids, especially in the case of chromosomal deficit (M4B) or chromosomal excess (Tri4B), did not show such significant changes compared with euploids. This relationship suggested that for triads, the perturbation of transcription induced by aneuploidy was largely buffered by the presence of more than one (highly similar) copy of the gene. The buffering effect was confirmed by the observation that differential transcription affected triads less frequently than it affected nontriad genes (Table 3). For instance, in the M4B vs euploid contrast, none of the 1748 homeologous group 4 chromosome triads showed evidence of differential transcription, whereas 738 (42.2%) of the genes mapped to chromosome 4B were classified as DTGs. An extra copy of chromosome 4B also had little effect on the abundance of transcripts generated by the triads. However, the two contrasts involving dosage of the chromosome arm 2AS segment (SegT2A vs euploid and N7B + SegT2A vs SegT2A) revealed quite a large number of differentially transcribed triads (DTTs). When a Gene Ontology (GO) analysis was conducted on these three sets of non-DTTs, it appeared that no specific GO term was significantly represented.

**Table 3:** DTGs belonging to a triad

Comparison	No. of expressed triads^*^	No. of DTGs^**^	No. of DTTs^***^
Euploid vs M4B	1748	738 (42.2%)	0
Euploid vs Tri4B	1748	172 (9.8%)	1 (0.06%)
Euploid vs SegT2A	455	225 (49.5%)	127 (27.9%)
SegT2A vs N7B + SegT2A	2102	/	987 (49.1%)

*, based on the varied chromosome/region; **, differentially transcribed genes (DTGs) in the varied chromosome/region; ***, differentially transcribed triads (DTTs) based on one-way ANOVA (*P* < 0.05)

**Fig. 4:**
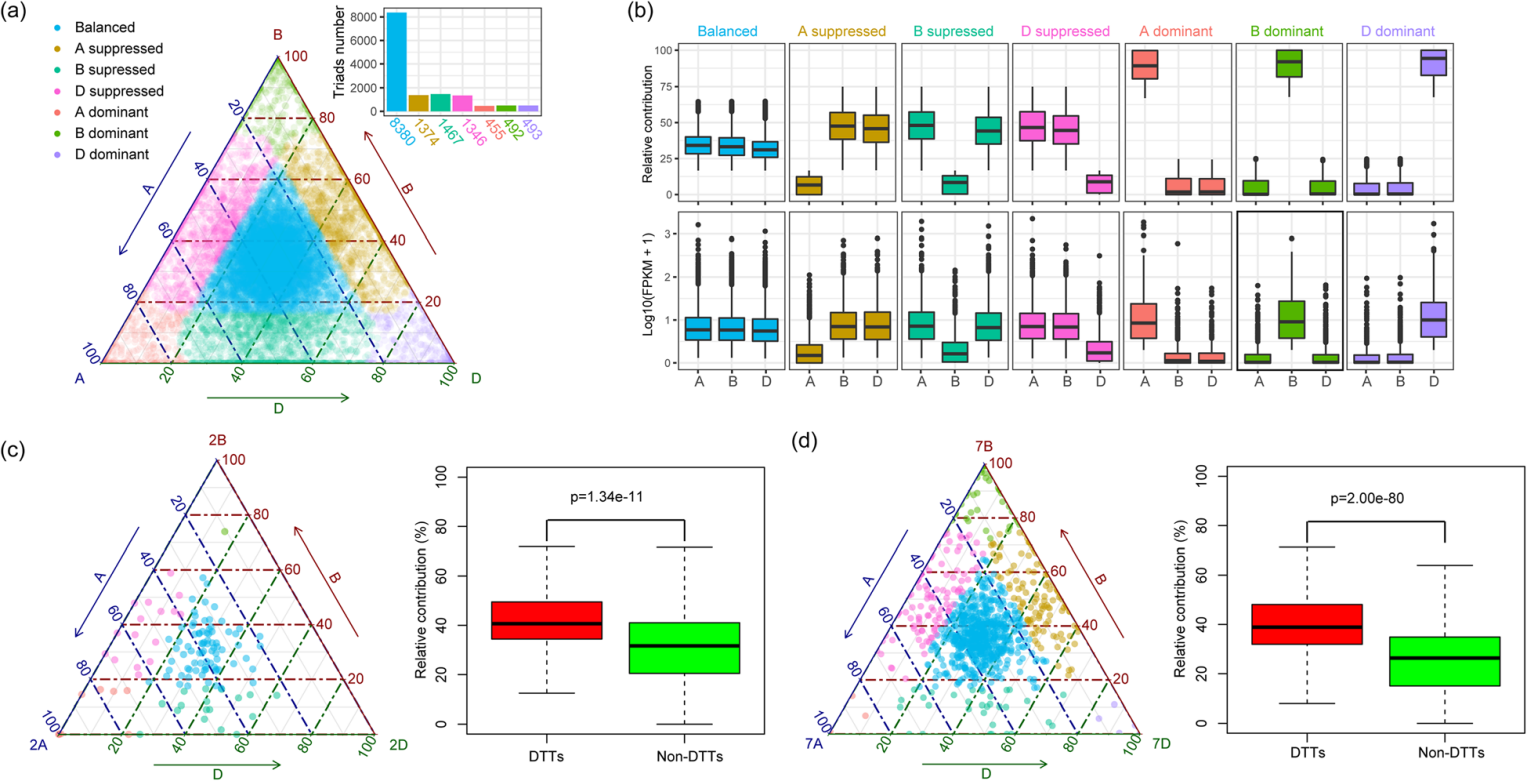
Bias in the transcription of homeoalleles mapped to the chromosomes involved in aneuploidy. (**a**) Ternary plot showing the transcript abundance of 14,007 triads (52,021 genes) in a euploid plant. Each circle represents a triad, with its A, B and D coordinates giving the relative contribution of each homeoallele. The distribution of the numbers of homeoallele transcription bias categories is shown at the top right. (**b**) Boxplots showing the relative contribution (upper panel) and FPKM distribution (lower panel) of each subgenome based on triad assignment to the seven categories. (c-d) The left panels show ternary plots illustrating the abundance of transcripts generated from triads exhibiting differential transcription identified from the contrasts (**c**) SegT2A vs euploid (127 triads) and (**d**) N7B + SegT2A vs SegT2A (987 triads); the right panel shows the relative contribution of homeoalleles in a euploid plant to the overall transcript abundance of both the DTTs and the non-DTTs. Student’s *t* test was used to determine statistical significance

Of the complete set of 18,474 triads present in the CS wheat genome, 75.8% (14,007) had an FPKM value above unity in the euploid sample. A ternary plot presenting the relative contribution of each of the homeoalleles making up the triads is shown in Fig. 4 a, and the whole set was classified into seven bias categories following the suggestion of Ramirez-Gonzalez et al. [24]. The “balanced” category, in which the three homeoalleles made equal contributions to the transcript, was represented by 59.8% of the triads, while the “single-homeoallele-suppressed” category was represented by 29.9% of the triads (the subgenome-origin distribution of the suppressed homeolog was A: 9.8%, B: 10.5%, and D: 9.6%). The “single-homeoallele-dominance” category was the least frequent (10.2%, with the subgenome-origin distribution of the dominant homeolog being A: 3.2% and B and D: both 3.5%). With respect to the seven transcription bias categories identified in euploid plants (Fig. 4 b), an overwhelming majority of the DTTs revealed by the SegT2A vs euploid contrast belonged to either the “balanced”, the “chromosome 2A dominant”, the “chromosome 2B suppressed” or the “chromosome 2D suppressed” category (Fig. 4 c). The DTTs identified in the N7B + SegT2A vs SegT2A contrast (Fig. 4 d) also featured strong transcription of 7B homeoalleles.

## Discussion

Allopolyploidization is an important driver of speciation in plants [25]. In general, natural allopolyploid species are genetically stable. However, nascent allopolyploids typically exhibit high degrees of cytogenetic, genetic and epigenetic instability [12, 26, 27], globally envisaged as a consequence of genomic shock [28]. Genetic discordance is also obvious in newly synthesized hexaploid wheat [29–31]. A recent comparison of transcriptomes revealed obvious differences in gene expression patterns between CS bread wheat and nascent SHW [24]. Such changes may also underlie divergence in regulatory mechanisms of gene expression between nascent and modern-day wheat. In the nascent wheat analysed here, aneuploidy had a greater effect on the transcription of genes on the varied chromosome (*cis*-effect) than on those mapped elsewhere (*trans*-effect). However, it is difficult to exactly investigate the transcriptomic impact of CS wheat aneuploidy since after over a half century of domestication, CS wheat-based aneuploid stocks harbour a wealth of chromosomal aberrations that can confound some experimental results arising from their use [32, 33]. Based on the results from several aneuploid lines of the CS wheat cultivar, there are more *trans*-effect genes than *cis*-effect genes [34]. This difference between nascent and modern-day wheat suggests that the expression regulation of some genes has been altered during the evolutionary process of bread wheat.

On the other hand, our observation of nascent wheat is consistent with early observations of CS wheat [5], in which monosomics and trisomics seem to be slightly abnormal, while nullisomics are greatly reduced in vigour and fertility. At the level of transcription, the behaviour of the present nascent wheat materials resembled that of the established wheat cultivar CS, where most triads show balanced transcription and homeoallele-dominant triads are rare [24]. Moreover, the three constituent subgenomes of hexaploid wheat are largely uncoupled at the transcriptional level [34]. In other words, homeologs do not differ significantly from other chromosomes in terms of their aneuploidy-induced effect on the transcription of the genes mapped to an unrelated chromosome. These similarities between nascent and modern-day hexaploid wheat indicate that the polyploid context and the allohexaploidization process itself play an important role in the gene transcription features of modern-day wheat.

A balanced dosage of gene products is required for a cell to perform as intended [1]. The consequences of an altered dosage, as induced by aneuploidy, can be cell-, tissue- and species-dependent. A common feature of many aneuploid conditions is a reduction in vigour and/or fertility [2, 4, 5], but compared to diploids, polyploids tend to exhibit a much higher tolerance to aneuploidy [5, 7–15]. Intuitively, the genetic buffer represented by the presence of multiple copies of the majority of genes in a polyploid should confer this tolerance. In hexaploid wheat, these genes are represented by triads, which appear to be transcriptionally highly stable, responding to a changed dosage of one of the homeoalleles by increasing/decreasing (as appropriate) the output of the other two; the result is that in both monosomic and trisomic lines, the abundance of a triad’s transcriptional product remains similar to that in the euploid (Table 3). The stability appears to break down when the number of chromosomes added or lost exceeds one, which may be one of the factors related to the phenomenon in which severe aneuploid states, such as nullisomy and tetrasomy, are rarely recovered among the progeny of newly synthesized hexaploid wheat [15]. In allotetraploid plants, such as those that arise in species belonging to the genera *Brassica* and *Tragopogon*, monosomic plants frequently require the presence of an extra homeolog for viability, while nullisomy needs to be compensated for by tetrasomy in the homeolog [7, 13].

The buffering effect of homeoalleles established in newly formed polyploids has evolutionary implications. The mutation of one homeoallele may have a subtle phenotypic effect. As a result, the selection pressure that acts to inhibit the transmission of newly formed alleles in nascent polyploids no longer applies to the same extent as in their corresponding diploid species [35, 36]. Thus, buffering is favourable for the rapid accumulation of genetic diversity. In concert, polyploids were more tolerant to mutations [36, 37]. Bread wheat evolved from a limited number of founder amphiploids, arising from recent allopolyploidization events occurring approximately 10,000 years ago. However, this crop has wide morphological variation and occupies a greater diversity of ecological niches and larger geographical areas than its diploid ancestors [29]. This success is related to the fact that its genome allows for rapid evolution [35].

SHW is an important resource for enhancing wheat yields across a diverse range of environments [19–21]. In addition to high diversity at the DNA level, SHW offers novel gene expression patterns compared to modern wheat [24, 38]. Additionally, the frequent production of aneuploids in SHW is favourable for manipulating chromosome dosage, which provides a breeding strategy for changing gene dosages. Many genes targeted in breeding are dosage sensitive. However, changes in whole chromosomes are often unfavourable for plant growth in breeding. Some aneuploids such as trisomics are unstable and will revert in a few generations to the disomic type [5]. One way of overcoming this problem is the use of segmental aneuploids that exhibit changed gene dosage but not an altered chromosome number. SegT2A, which harbours four copies of a segment of chromosome arm 2AS, is such a segmental aneuploid. SegT2A plants produce larger spikes than euploid plants. The use of SegT2A in wheat breeding is ongoing.

The tolerance of allopolyploid plants to aneuploidy provides a convenient model with which to study how gene interactions are altered by changes in gene dosage. The current study has suggested a number of ways in which homeoallele transcription is regulated, and the insights gained may prove informative in understanding allopolyploid genome evolution and in progressing the breeding of polyploid crop species.

## Conclusions

In the present investigation, we performed transcriptome analysis of four aneuploid lines spontaneously generated from synthetic hexaploid wheat that represents primitive bread wheat at the early generation of ancestral allohexaploidization events. We found that most genes within affected chromosomes were transcribed at a level proportional to their copy number. Aneuploidy had a greater effect on the strength of transcription of genes mapped to the chromosome present in a noneuploid dose than on that of genes mapped elsewhere in the genome. Moreover, aneuploidy has no biased effects across homoeologous groups. Our experiments revealed a prominent phenomenon termed homoeolog expression buffering, wherein changes in a copy of homoeologs have little effect on the total expression of all homoeologs. This mechanism can maintain the total expression dosage balance of duplicated genes and hence can be used to explain why allopolyploid plants are highly tolerant to aneuploidy, especially when one chromosome is lost (monosomy) or gained (trisomy). Our findings shed new light on the genetic regulation of homoeologs and have implications for understanding allopolyploid genome evolution and crop breeding.

## Methods

### Plant materials

The aneuploid individuals used arose from the selfed progeny of an SHW derived from a hybrid formed between the tetraploid subspecies *Triticum turgidum ssp. turgidum* AS313 (containing the A and B subgenomes of hexaploid wheat) and the diploid *Ae. tauschii* ssp. *tauschii* AS60 (D subgenome). The initial doubled allohaploid (S1 generation) was allowed to self-pollinate to produce the S2 generation. The selected aneuploid individuals were S3 segregants bred from two S2 plants; the chromosomes involved in the aneuploidy were the entire 4B and 7B chromosomes and a segment of the short arm of chromosome 2A (Fig. 1) [39]. The chromosomal constitution of the selections was monitored by karyotyping squashes of seedling root tips. The selected plants were subjected to RNA-Seq, and their phenotypes were monitored following their planting in the field (Wenjiang Experimental Station of Sichuan Agricultural University, 30°36′N, 103°41′W) during the 2014–2015 crop season. All of the plant materials used in this study were synthesized and maintained by the Triticeae Research Institute of Sichuan Agricultural University. The formal identification of the aneuploid plants was conducted by the corresponding author of this article.

### Karyotyping

The protocols used to process root tip samples and to perform multicolour FISH followed those described by Komuro et al. [40] and Zeng et al. [39]. The oligonucleotide probes Oligo-pSc119.2 and Oligo-pTa535 were synthesized by TSINGKE Biological Technology Company (Beijing, China) and were 5′ end labelled with either TAMRA or 6-FAM. Genomic in situ hybridization (GISH) experiments were performed as described by Hao et al. [41]. To distinguish between the chromosome complements of each of the three wheat subgenomes, total genomic DNA extracted from the A genome donor *Triticum urartu* and the D genome donor *Ae. tauschii* was labelled with digoxigenin-11-dUTP and biotin-16-dUTP (Roche Diagnostics GmbH, Mannheim, Germany), respectively. Unlabelled genomic DNA from *Aegilops speltoides* (putative B genome donor) was used for blocking.

### Sample preparation and transcriptome sequencing

Whole spikes harvested at the heading stage were snap frozen in liquid nitrogen and held at − 80 °C until required. Total RNA was extracted from the frozen tissue using an RNAprep Pure Plant kit (TIANGEN, Beijing, China) according to the manufacturer’s instructions. The integrity and quality of the resulting RNA were assessed using a 2100 Bioanalyzer (Agilent Technologies, Palo Alto, CA, USA). Transcriptome libraries were constructed using the NEBNext® Ultra™ RNA Library Prep Kit for Illumina (New England Biolabs, USA) following the manufacturer’s recommendations. The libraries were sequenced using a HiSeq 2000 platform (Illumina, San Diego, CA, USA) following standard protocols. Contaminant and low-quality reads were discarded by imposing a Q30 threshold of 80% and a maximum of 0.2% ambiguous base calls.

### Read alignment and RNA-Seq data analysis

TopHat v2.0.11 software (http://ccb.jhu.edu/software/tophat/index.shtml) was used to align the set of clean reads against the CS bread wheat reference genomic sequence (urgi.versailles.inra.fr/download/iwgsc/IWGSC_RefSeq_Assemblies/v1.0), allowing a maximum of one mismatch per alignment (parameters: bowtie1 -N 1 -r 40 --library-type fr-unstranded). To avoid biased expression estimates due to spurious assignments of transcriptome sequences to the incorrect wheat genome, the “accepted_hits.bam” files returned by TopHat were filtered as described by Pfeifer et al. [42]. Raw read counts of genes for each sample were obtained based on the GFF file and bam files using the HTSeq-count tool from the Python package HTSeq [43]. Transcript abundances were estimated from the FPKM values [44]. Pearson’s correlation analysis was conducted between the three or four individual plants used as biological replicates by using the “cor” function based on log_2_ (FPKM + 1)-transformed data [42]. DTGs were identified after normalization of raw read counts using the DESeq2 program [45]. Transcripts associated with an FDR-adjusted *P* value [46] < 0.05 were considered to exhibit a statistically significant difference in transcript abundance. A GO enrichment analysis was performed using the OmicShare platform (www.omicshare.com/tools/Home/Report/goenrich). The wheat GO annotation file was downloaded from the URGI site (urgi.versailles.inra.fr/download/iwgsc/IWGSC_RefSeq_Annotations/v1.0/).

### Assessment of differences in transcript abundance

The observed FPKM values for each transcript were averaged across the three or four replicate samples of each genotype. Genes for which an FPKM value greater than unity was recorded in at least one of the genotypes were included in the calculation of ratios between the aneuploid and euploid FPKM values. The log_2_ values of these ratios were plotted with respect to the genes’ physical position using the R package ggplot2 v3.1.0 [47]. Simultaneously, the loess function was applied with a smoother span (bin width) of 0.1 in R to smooth the distribution of the ratios along the length of the chromosome.

### The effect of chromosomal dosage on transcript abundance

A Pearson correlation test was performed to test whether each transcript’s abundance (only those genes for which an FPKM value greater than unity was recorded in at least one of the genotypes were included) was related to the dosage of the chromosome on which it was located [48]. Each replicate of a given genotype was considered an independent experimental unit. The resulting *P* values were FDR adjusted using the adjustment method in R. The FDR threshold for considering the correlation between gene dosage and transcript abundance was set to 0.05.

### Transcription profiling of members of homeoallelic sets

The analysis focused on a set of 55,422 genes represented as sets of three homeoalleles (“triads”): the genes represented in 17,400 of the triads mapped to a syntenic region in each of the three wheat subgenomes, while those represented in 1074 triads mapped to nonsyntenic regions [24]. To allow the analysis to include triads in which only one of the homeoalleles was transcribed, a triad was assumed to be transcribed when the summed FPKM values of the three homeoalleles were greater than unity. The FPKM value of each triad was used as the denominator to standardize the relative transcript abundance of each homeoallele. The relative contribution of each homeoallele was plotted in the form of a ternary diagram using the R package ggtern v3.1.0 (www.ggtern.com). Fold changes between genotypes were calculated and visualized using the R package ggplot2 v3.1.0 [47]. The FDR-adjusted *P* threshold for determining the significance of triad transcript abundance was set to 0.05, based on a one-way ANOVA implemented in R.

### The phenotypic consequences of aneuploidy

The length and width of each of the first three seedling leaves were measured when the leaves were fully expanded. Plant height was considered the height of the longest tiller, and both spike length and spikelet number were measured from this tiller. Fertility was assessed on the basis of only the primary and secondary florets.

### Statistical analyses

All statistical analyses were performed using programs implemented in either R v3.4.4 software or Microsoft Excel 2007.

## Supplementary information

**Additional file 1 **: **Fig. S1** FISH karyotypes of the newly synthesized hexaploid wheat. (a) Euploid plant with 2n = 42. (b) M4B: plant lacking one copy of chromosome 4B (red arrow). (c) Tri4B: plant carrying an extra copy of chromosome 4B (red arrow). (d) SegT2A: plant harbouring a duplicated segment of the short arm of chromosome 2A (red arrow). (e) N7B + SegT2A, plant lacking both copies of chromosome 7B and harbouring a duplicated segment of the short arm of chromosome 2A (red arrow). **Fig. S2** FISH- and GISH-based karyotyping used to validate the aneuploid status of the SegT2A material. (a) FISH karyotype of an SHW line carrying a wild-type copy of chromosome 2A; (b) FISH karyotype of an SHW line carrying the version of chromosome 2A (arrowed) shown to include a duplication of a part of its short arm. (c) GISH karyotype of an SHW line carrying SegT2A. The karyotype suggests that the copy of chromosome 2A contains additional sequences inherited from an A-subgenome chromosome. Labelling based on the probes Oligo-pSc119.2 (red) and Oligo-pTa-535 (green). **Table S1** The effect of aneuploidy on leaf length and width. *, **: means differ from one another at *P* ≤ 0.05 and ≤ 0.01, respectively (statistical significance was determined using Student’s *t* test). Comparisons were made between euploid plants and the aneuploids M4B, Tri4B and SegT2A and between N7B + SegT2A and SegT2A. **Table S2** RNA-Seq reads and their alignment with the CS wheat reference genome sequence v1.0. ^a^ Uniquely aligned reads designated according to the criteria suggested by Pfeifer et al. [42]. **Table S3** GO analysis of DTGs mapped to chromosomes not involved in aneuploidy.

## Data Availability

All data generated or analysed during this study are included in this published article and its supplementary information files. The RNA-seq data generated by this study have been deposited in the National Center for Biotechnology Information Sequence Read Archive (accession no. PRJNA607628).

## Abbreviations

CS: Chinese Spring
DTGs: Differentially transcribed genes
DTTs: Differentially transcribed triads
FISH: Fluorescent in situ hybridization
FPKM: Fragments per kilobase of gene per million mapped reads
GISH: Genomic in situ hybridization
GO: Gene Ontology
M4B: Lacking one copy of chromosome 4B
N7B + SegT2A: Lacking both copies of chromosome 7B, along with the same altered version of chromosome 2A carried by SegT2A
SegT2A: Harbouring four copies of a segment of chromosome arm 2AS
SHW: Synthetic hexaploid wheat
Tri4B: Trisomic for chromosome 4B
