# Supplementary material for: A transcriptomic view of the ability of nascent hexaploid wheat to tolerate aneuploidy
Source: BMC Plant Biol. 2020 Mar 4;20:97. doi: 10.1186/s12870-020-2309-6 (PMC7057484; doi:10.1186/s12870-020-2309-6)
Supplement: Supplementary file 1 — Additional file 1 : Fig. S1 FISH karyotypes of the newly synthesized hexaploid wheat. (a) Euploid plant with 2n = 42. (b) M4B: plant lacking one copy of chromosome 4B (red arrow). (c) Tri4B: plant carrying an extra copy of chromosome 4B (red arrow). (d) SegT2A: plant harbouring a duplicated segment of the short arm of chromosome 2A (red arrow). (e) N7B + SegT2A, plant lacking both copies of chromosome 7B and harbouring a duplicated segment of the short arm of chromosome 2A (red arrow). Fig. S2 FISH- and GISH-based karyotyping used to validate the aneuploid status of the SegT2A material. (a) FISH karyotype of an SHW line carrying a wild-type copy of chromosome 2A; (b) FISH karyotype of an SHW line carrying the version of chromosome 2A (arrowed) shown to include a duplication of a part of its short arm. (c) GISH karyotype of an SHW line carrying SegT2A. The karyotype suggests that the copy of chromosome 2A contains additional sequences inherited from an A-subgenome chromosome. Labelling based on the probes Oligo-pSc119.2 (red) and Oligo-pTa-535 (green). Table S1 The effect of aneuploidy on leaf length and width. *, **: means differ from one another at P ≤ 0.05 and ≤ 0.01, respectively (statistical significance was determined using Student’s t test). Comparisons were made between euploid plants and the aneuploids M4B, Tri4B and SegT2A and between N7B + SegT2A and SegT2A. Table S2 RNA-Seq reads and their alignment with the CS wheat reference genome sequence v1.0. a Uniquely aligned reads designated according to the criteria suggested by Pfeifer et al. [42]. Table S3 GO analysis of DTGs mapped to chromosomes not involved in aneuploidy. [file 12870_2020_2309_MOESM1_ESM.docx]

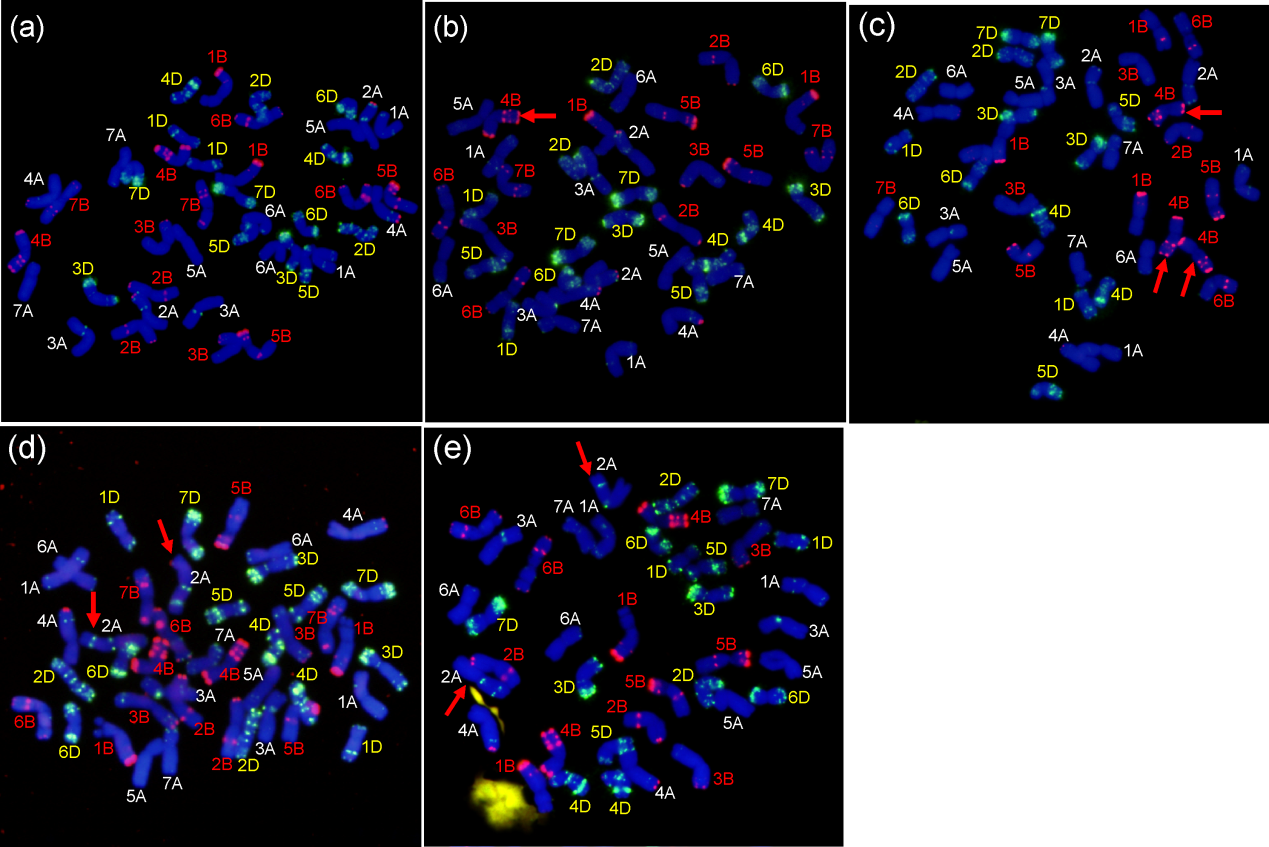


**Fig. S1 FISH karyotypes of the newly synthesized hexaploid wheat.** (a) Euploid plant with 2n=42. (b) M4B: plant lacking one copy of chromosome 4B (red arrow). (c) Tri4B: plant carrying an extra copy of chromosome 4B (red arrow). (d) SegT2A: plant harboring a duplicated segment of the short arm of chromosome 2A (red arrow). (e) N7B+SegT2A, plant lacking both copies of chromosome 7B and harboring a duplicated segment of the short arm of chromosome 2A (red arrow).

**Fig. S2 FISH- and GISH-based karyotyping used to validate the aneuploid status of the SegT2A material.** (a) FISH karyotype of a synthetic hexaploid wheat carrying a wild type copy of chromosome 2A; (b) FISH karyotype of a synthetic hexaploid wheat carrying the version of chromosome 2A (arrowed) shown to include a duplication of a part of its short arm. (c) GISH karyotype of a synthetic hexaploid wheat carrying SegT2A. The karyotype suggests that the copy of chromosome 2A contains additional sequences inherited from an A sub-genome chromosome. Labeling based on probes Oligo-pSc119.2 (red) and Oligo-pTa-535 (green).

**Table S1 The effect of aneuploidy on leaf length and width.**

| Genotype | No. of plants | 1th | | 2th | | 3th | |
| --- | --- | --- | --- | --- | --- | --- | --- |
|  |  | Length | Width | Length | Width | Length | Width |
| M4B | 10 | 13.96±0.94 | 0.39±0.02 | 16.11±1.3 | 0.49±0.02 | 15.36±1.14 | 0.65±0.02^*^ |
| Euploid | 28 | 12.95±0.61 | 0.42±0.01 | 15.07±0.59 | 0.52±0.01 | 13.77±0.54 | 0.71±0.01 |
| Tri4B | 5 | 10.9±0.73 | 0.42±0.02 | 12.87±1.31 | 0.52±0.05 | 11.65±1.27 | 0.66±0.04 |
| SegT2A | 5 | 13.66±0.95 | 0.44±0.02 | 16.05±0.9 | 0.51±0.004 | 15.18±0.96 | 0.74±0.02 |
| N7B+SegT2A | 11 | 7.61± 0.47^**^ | 0.39± 0.01^**^ | 9.85± 0.62^**^ | 0.4± 0.01^**^ | 10.25±0.75^**^ | 0.51± 0.01^**^ |

*, **: means differ from one another at, respectively, P ≤0.05 and ≤0.01 (statistical significance was determined using the Students’ *t* test). The comparisons were made between euploid plants and the aneuploids M4B, Tri4B and SegT2A, and between N7B+SegT2A and SegT2A.

**Table S2 RNA-Seq reads and their alignment with the reference genome sequences v1.0 of Chinese Spring wheat.**

| Sample | Total bases | Total Reads | Uniquely aligned reads^a^ | Ratio (%) | Correlation coefficient |
| --- | --- | --- | --- | --- | --- |
| Euploid.1 | 2368510701 | 46901202 | 38963178 | 83.08 | 0.98 (0.98-0.98) |
| Euploid.2 | 2049719149 | 40588498 | 33027078 | 81.37 |  |
| Euploid.3 | 2169226086 | 42954972 | 35476586 | 82.59 |  |
| M4B.1 | 3087486574 | 61138348 | 48916278 | 80.01 | 0.93 (0.87-0.97) |
| M4B.2 | 2566815818 | 50828036 | 42209198 | 83.04 |  |
| M4B.3 | 2668845109 | 52848418 | 43882244 | 83.03 |  |
| M4B.4 | 2456472207 | 48643014 | 40398348 | 83.05 |  |
| Tri4B.1 | 2177445062 | 43117724 | 34470570 | 79.95 | 0.89 (0.85-0.96) |
| Tri4B.2 | 2166727144 | 42905488 | 35197088 | 82.03 |  |
| Tri4B.3 | 2593954013 | 51365426 | 42836936 | 83.40 |  |
| Tri4B.4 | 2301311058 | 45570516 | 37338400 | 81.94 |  |
| SegT2A.1 | 2509057352 | 49684304 | 40400206 | 81.31 | 0.97 (0.96-0.99) |
| SegT2A.2 | 3191136511 | 63190822 | 50460364 | 79.85 |  |
| SegT2A.3 | 2302709403 | 45598206 | 37740382 | 82.77 |  |
| N7B+SegT2A.1 | 2145438465 | 42483930 | 34647698 | 81.55 | 0.96 (0.93-0.99) |
| N7B+SegT2A.2 | 2037886494 | 40354188 | 33392912 | 82.75 |  |
| N7B+SegT2A.3 | 2205152493 | 43666386 | 34414716 | 78.81 |  |
| N7B+SegT2A.4 | 2598397609 | 51453418 | 42861220 | 83.30 |  |

^a^ Uniquely aligned reads designated according to the criteria suggested by Pfeifer et al. (2014).

**Table S3 GO analysis of DTGs mapping to chromosomes not involved in aneuploidy.**

| Genotype | Regulation | GO Term | Description | Ontology | FDR |
| --- | --- | --- | --- | --- | --- |
| N7B+SegT2A | Down | GO:0055114 | oxidation-reduction process | BP | 7.79E-09 |
|  |  | GO:0015979 | photosynthesis | BP | 7.33E-08 |
|  |  | GO:0006721 | terpenoid metabolic process | BP | 4.92E-05 |
|  | Up | GO:0055114 | oxidation-reduction process | BP | 4.30E-04 |
|  |  | GO:0006950 | response to stress | BP | 6.03E-04 |
|  |  | GO:0009266 | response to temperature stimulus | BP | 4.04E-03 |
| M4B | Down | GO:0016702 | oxidoreductase activity, acting on single donors with incorporation of molecular oxygen, incorporation of two atoms of oxygen | MF | 2.83E-05 |
|  |  | GO:0033897 | ribonuclease T2 activity | MF | 2.83E-05 |
|  |  | GO:0003993 | acid phosphatase activity | MF | 8.25E-04 |
| SegT2A | Down | GO:0055114 | oxidation-reduction process | BP | 7.62E-03 |
|  |  | GO:0043169 | cation binding | MF | 6.24E-04 |
|  |  | GO:0030532 | small nuclear ribonucleoprotein complex | CC | 1.72E-02 |
|  | Up | GO:0055114 | oxidation-reduction process | BP | 4.88E-02 |
|  |  | GO:0046914 | transition metal ion binding | MF | 1.16E-03 |
|  |  | GO:0005506 | iron ion binding | MF | 5.98E-03 |
